# Editorial: Vagus nerve-mediated drive in supporting homeostasis: optimizing global health through monitoring and stimulating vagal function

**DOI:** 10.3389/fphys.2023.1279258

**Published:** 2023-09-06

**Authors:** Claire Marie Rangon, Adam Niezgoda, Emmanuel Moyse, Stephen W. Porges

**Affiliations:** ^1^ Child Neurologist and Pain Specialist, INWE’CARE Medical Center, Saint-Cloud, France; ^2^ Department of Neurology, Heliodor Swiecicki University Hospital, University of Medical Sciences of Poznań, Poznań, Poland; ^3^ INRAE Centre Val-de-Loire, Physiology of Reproduction and Behavior Unit (PRC UMR-85), Nouzilly, France; ^4^ Traumatic Stress Research Consortium, Kinsey Institute, Indiana University, Bloomington, IN, United States; ^5^ Department of Psychiatry, University of North Carolina at Chapel Hill, Chapel Hill, NC, United States

**Keywords:** noninvasive vagus nerve stimulation, prevention, epigenetics, heart rate variability, personalised medicine

## Introduction

Epigenetics encompasses the changes in gene expression triggered by environmental cues that occur without altering the underlying DNA sequences. Although epigenetics involves a limited number of identified mechanisms so far (DNA methylation, histone modifications and noncoding RNAs), the field of Epigenetics is spreading rapidly over integrated physiopathology (Jeffries, 2020). The importance of epigenetics has been acknowledged in cancer (Sun et al., 2022), sepsis (Binnie et al., 2020), autoimmune/inflammatory diseases (Surace and Hedrich, 2019), addiction (Hamilton and Nestler, 2019), aging (Pal and Tyler, 2016; Pérez et al., 2022), neurodegenerative diseases (Berson et al., 2018; Zhang et al., 2022) and even in neurodevelopmental (Esposito et al., 2018; Wu et al., 2020) and psychiatric diseases (Abdolmaleky et al., 2023). Likewise, a broad and expanding set of therapies in clinics have been potentialized by tuning the vagal complex of brainstem, i.e., the key neural node of homeostasis, *via* non-invasive Vagus Nerve Stimulation (VNS) (Hilz, 2022). Moreover, Heart Rate Variability (HRV), a physiologically validated and easily accessible read-out of the vagal tone (Lewis et al., 2012), is acknowledged as a polyvalent prognostic tool (Gidron et al., 2018; Mol et al., 2021). This is not surprising, since Epigenome is considered as a “mediator for host-microbiome crosstalk” (Peery et al., 2021) and since vagus nerve represents the fastest pathway of the Microbiota-Gut-brain Axis (Fülling et al., 2019). However, except for regulating neuro-inflammation (Chen et al., 2022), the mechanisms through which VNS influences peripheral and central nervous system plasticity are not well described yet, limiting therapeutic optimization (Morrison et al., 2020; Keute and Gharabaghi, 2021). Therefore, as the vagal complex (i.e., peripheral and brainstem components) is definitely prone to bring about epigenetic modulations, notably through neuro-endocrine stimuli (Gil et al., 2013), we questioned the epigenetic role of vagus nerve, in order to promote noninvasive VNS, guided by HRV monitoring, as a universal therapy to stay healthy.

Given the lack of evidence in the mentioned areas, this Research Topic aimed to.

1) update the key role of the brainstem vagal center in global homeostasis and health, and its position as bidirectional interface for neuro-immune interactions (bottom-up and top-down) in both animal models and clinical studies.
2) Assess the epigenetic mechanisms mediating adaptations of homeostatic reflexes and HRV inside the vagal complex of brainstem with focus on the differential effects of electrical parameters used.
3) Draw a research roadmap for assessing VNS as a putative efficient preventive therapy for improving health during an extended span.

## A summary of the article contribution

This Research Topic gathered four articles, two original research study in Humans (Maharjan et al.; Konakoglu et al.), one original research study in a rat model (Carnevali et al.) and one review. HRV was specifically assessed in two studies (Maharjan et al.; Carnevali et al.).

The first study by Maharjan et al. documents the use of olfactory stimulation, instead of the more prevalent use of electrical stimulation, as a method of vagal nerve neuromodulation. Their paper illustrates the potent impact of olfactory stimulation in mediating autonomic changes. Their study is the first to establish a dose- and duration-specific effects of odors on the human Autonomic Nervous System (ANS). This study illustrates that there are other pathways of neuromodulation that may involve sensory stimuli that are not solely traveling through afferent pathways that directly influence the vagus that have frequently defined the domain of VNS. Their study has conceptual features similar to earlier studies applying other sensory stimuli such as music that has been interpreted as an “acoustic” vagal nerve stimulation (Porges et al., 2014; Rajabalee et al., 2022). Interestingly, this olfactive neuromodulation happens to impact specifically the vagal component of the ANS (but not the sympathetic one), suggesting that the vagal response profiles may be more modifiable and thus more sensitive to epigenetic factors than the sympathetic branch of the ANS, which may be linked to foundational survival circuits and less malleable. The flexibility of this “olfaction-autonomic state” circuit was also influenced by sex hormonal status (gender and menstrual stage matter).

In line with this latter finding, the second study by Carnevali et al. highlights the importance of considering biological sex in the analysis and interpretation of HRV data in rats and in other mammals including humans. Moreover, because of the importance of the body size on cardiac chronotropy, this study shows a contrast between the neurometric HRV and the allometric heart rate.

The third study by Williamson et al., advocates for noninvasive VNS to counteract the epigenetics of brain aging without causing side effects in this vulnerable population. The main putative mechanisms involved are reviewed (notably neurotransmitter and functional connectivity changes besides control of inflammation) as well as vagus nerve changes with age, in order to delay the onset of neurodegenerative diseases, thanks to optimized noninvasive VNS protocols in addition to existing behavioral interventions.

The final paper by Konakoglu et al. focuses on the bidirectional effect of auricular vagus nerve stimulation, challenging future VNS clinical trials to assess efficiency at both peripheral and central levels. Moreover, the critical Research Topic of the targeting of the proper ear to stimulate (right or left) is assessed in this study, knowing that noninvasive VNS has been currently administered in a unilateral-left fashion (Badran et al., 2019). Interestingly, Konakoglu et al. findings do not support this left-only convention, as another recent paper (Peng et al., 2023), both suggesting that the choice of the ear should depend mainly on the localization of the targeted lesions (either brain or body lesions) to treat.

## Conclusion

Epigenetics provides a revolutionary perspective on the dynamic nature of gene expression and a potential for recovery. The four articles support vagus nerve as a pivotal epigenetic transducer, sensitive to several modifications of the environment of the host (like odors and the localization of the stimulation on the body) or of the host itself (age and hormonal status), impacting both central (EEG modification) and peripheral nervous system (wrist extension muscles modification), thus confirming its bidirectional role (bottom-up and top-down). But above all, they enlighten the roadmap for future VNS clinical trials, by emphasizing the key role of sex and age Research Topic of the host, as well as the choice of the nature of stimulation applied to the vagus nerve and proper ear targeting, opening new avenues for personalized medicine.

Moreover, unraveling the role of VNS on genetic expression is likely to allow valuable insights into optimizing the complex interplay between nature and nurture, challenging the notion of predetermined destiny thanks to noninvasive preventive therapeutic interventions. Last but not least, the knowledge gained may also ensure a more equitable access to healthcare, considering the vast potential of portals to stimulate vagal function, including an olfactive neuromodulation in case of headache, for instance. Thus, as suggested by the polyvagal Theory, vagus-nerve-mediated drive of homeostasis will ultimately support mammalian sociality with all it biobehavioral benefits (Porges, 2021; Porges, 2023).
